# Pericardial calcification: A case report of a three-dimensional disease

**DOI:** 10.1016/j.ijscr.2019.03.009

**Published:** 2019-03-19

**Authors:** Raja Ohri, Kareem Salhiyyah, Stephen Harding, Sunil Ohri

**Affiliations:** Wessex Cardiothoracic Centre, University Hospitals Southampton, Tremona Road, Southampton, SO16 6YD, United Kingdom

**Keywords:** Constrictive pericarditis, Pericardial calcification, Three-dimensional imaging

## Abstract

**Introduction:**

Constrictive pericarditis is an important cause of diastolic heart failure. The relationship between the presence and degree of pericardial calcification with constrictive pericarditis is variable, however this should be an early warning sign to initiate appropriate investigations.

**Presentation of case:**

A 64-year-old gentleman presented with dyspnoea and dizziness on exertion. Plain posterior-anterior chest X-ray showed mild to moderate pericardial calcification. He had an episode of atrial fibrillation which resolved spontaneously and thought to be the cause of his symptoms. Patient symptoms progressed. Further investigations including CT scan confirmed extensive constrictive pericarditis. He underwent a successful percardiectomy and full recovery.

**Discussion:**

Constrictive pericarditis can be the consequence of different conditions such as tuberculosis, radiation, idiopathic or many others. Pericardial calcification is present in less than 25% of all cases of constrictive pericarditis, and patient with it are prone to develop atrial fibrillation. The presence of pericardial calcification on plain chest radiograph is an important indicator for the possibility of pericardial dysfunction. Many cases of pericardial calcification are benign without any clinical significance; however three-dimensional imaging is required to determine the calcium load and aid in reaching appropriate diagnosis.

**Conclusion:**

Chest X-ray is valuable detector of pericardial calcification, a surrogate of pericardial constriction, but it is important to remember that pericardial calcification is a three-dimensional condition, and should be further investigated by lateral chest X-ray, CT scan and then appropriate functional imaging.

## Highlights

• Constrictive pericarditis is an important cause of diastolic heart failure.
• Pericardial calcification is present in less than 25% of all cases of CP.
• Chest X-ray is valuable detector of pericardial calcification, a surrogate of pericardial constriction.
• Pericardial calcification is a three-dimensional condition, and should be investigated by lateral CXR, CT scan and appropriate functional imaging.

## 1 Introduction

Constrictive pericarditis (CP) is a relatively uncommon condition caused by pericardial dysfunction secondary to different aetiologies. It results in impaired cardiac function and associated with diastolic heart failure [1]. It is associated with high morbidity and mortality and the early diagnosis and intervention improve outcomes [2]. The diagnosis can be challenging and require high index of suspicion. The relationship between pericardial calcification and CP is not definite. The presence of some pericardial calcification can be a benign phenomenon without any functional effect. On the other hand, pericardial calcification is only present is less than quarter of the cases of CP [3].

In this report, we present a case of CP associated with mild to moderate pericardial calcification, and emphasize on the importance of three-dimensional imaging in assessing the true extent of calcification and getting the accurate diagnosis. This report was written in line with the SCARE criteria [4].

## 2 Case presentation

**Fig. 1:**
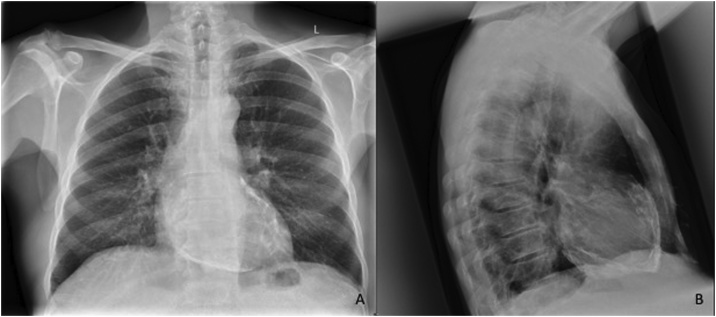
(A): PA Chest X-ray showing mild to moderate pericardial calcification (B): Lateral Chest X-ray showing more extensive pericardial calcification.

**Fig. 2:**
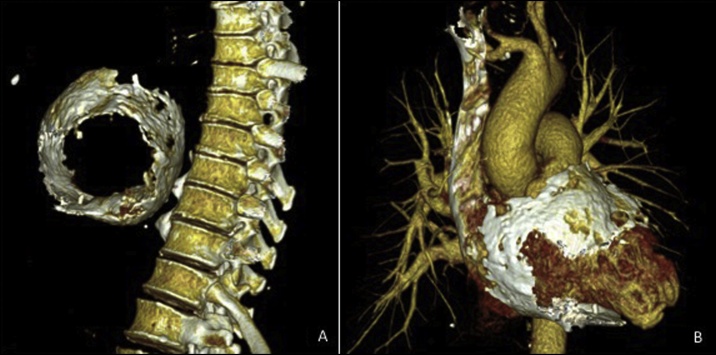
(A): Three-dimensional CT reconstruction showing extensive circumferential pericardial calcification (B): Three-dimensional CT reconstruction with soft-tissue extraction showing extensive circumferential pericardial calcification.

**Fig. 3:**
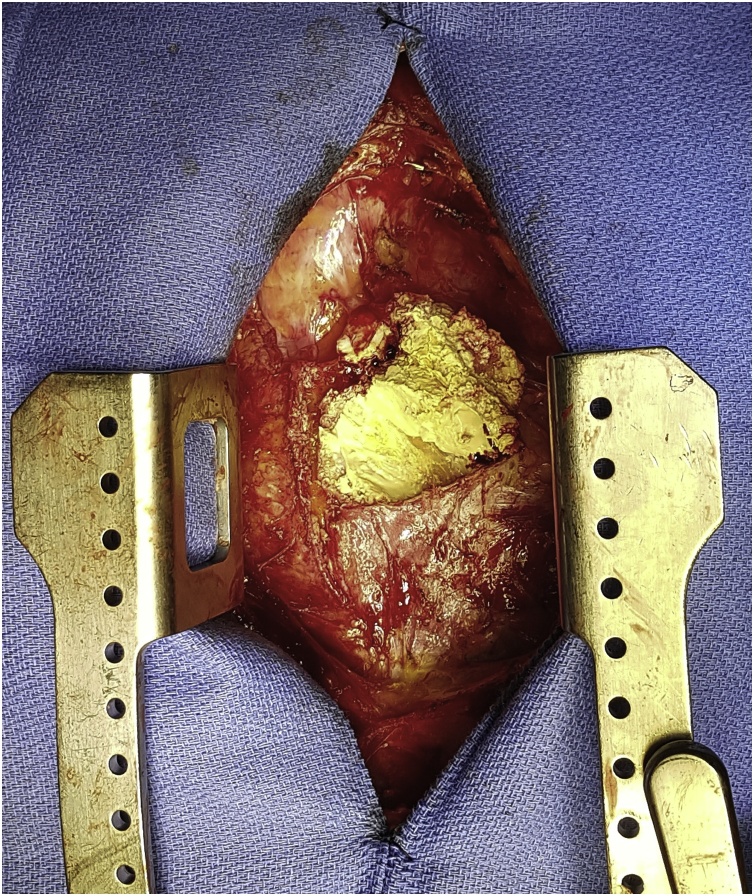
Intra operative image showing severe pericardial calcification.

A 64-year-old gentleman previously fit and well, apart from mild hypertension, generally active and enjoyed running, presented to the emergency department (ED) with an episode of acute shortness of breath and dizziness after running. He described episodes of progressive breathlessness over the previous few weeks. He was found to have atrial fibrillation (AF) which resolved spontaneously. The diagnosis of paroxysmal AF was made and his symptoms were attributed to his dysrhythmia. CT brain was normal, but a plain PA chest X-ray showed mild to moderate mediastinal calcification. The patient was discharged from ED without further treatment. However, he continued to deteriorate with progressive breathlessness and limitation of exercise tolerance. Eventually he was referred to a cardiologist and diagnosed with CP. The diagnosis was confirmed mainly on MRI scan which showed pericardial thickening. The interventricular septal morphology was abnormal with bowing into left ventricle consistent with ventricular coupling. This is exaggerated during dynamic inspiration and normalises during expiration consistent with constrictive physiology. Subsequently the patient was referred for surgery (Fig. 1, Fig. 2, Fig. 3).

He underwent successful percardiectomy. The pericardium was stripped from the surface of the heart anteriorly, laterally from phrenic to phrenic and inferiorly. The right atrium was also released up to and around both cavae. He had an uneventful post-operative recovery. He was extubated 6 h post op. He intensive care stay was 40 h, during which he was kept on small dose dopamine (1.3 mcg/kg/min) as a precaution. His hospital length of stay was 5 days. He has returned to full activity and remains asymptomatic.

## 3 Discussion

Constrictive pericarditis is an important cause of diastolic heart failure. It is characterized by focal or global loss of pericardial compliance. It is associated with a variable degree of thickening, fibrosis and calcification [1]. Tuberculosis (TB) remains one of the leading causes in the developing world, whereas idiopathic or viral CP are more common in developed countries. Other causes of CP including radiotherapy, post-cardiac surgery and many other aetiologies.

Pericardial calcification is present in less than 25% of all cases of CP [3]. The cause of pericardial calcification is usually of uncertain aetiology. CP usually presents with symptoms of right heart failure. Patients with CP have increased incidence of developing atrial fibrillation [5].

The diagnosis of CP is made through a combination of different investigations, including echocardiography, computerised tomography (CT), magnetic resonance imaging (MRI) and right heart catheterisation [1,6]. Although chest X-ray has limited value in the diagnosis of CP, it has an important role in detecting pulmonary or pleural diseases and localising calcification to the pericardium.

This patient presented to the ED with progressive shortness of breath on exertion. He was found to have paroxysmal atrial fibrillation. A plain PA chest X-ray (CXR) film showed mild to moderate calcification, but a lateral chest X-ray was not undertaken. The symptoms were attributed to the AF alone. Unfortunately, no further investigations were organised. The pericardial calcification present on his PA CXR was overlooked. As the patient continued to deteriorate, he was only then referred to a cardiologist.

Early intervention with pericardiectomy is a predictor of good early and late outcome in CP [2]. Prolonged constriction can result in myocardial atrophy, residual constriction, and persistent heart failure despite successful pericardiectomy.

## 4 Conclusion

Although CP is an uncommon condition, this diagnosis should be kept in mind when patients present with cardiac symptoms. Chest X-ray remains a valuable early detector of pericardial calcification and hence increases the likelihood of CP, which should trigger a lateral CXR film and then appropriate subsequent functional imaging to confirm or refute the suspected diagnosis [6]. A prompt diagnosis and intervention will improve outcome.

## Conflicts of interest

No conflict of interest.

## Funding

No funding.

## Ethical approval

No ethical approval was required.

## Consent

Patient has kindly given a written informed consent for publication which is available for the Editor-in-Chief upon request.

## Author contribution

**Raja Ohri:** Resources, Software, Methodology, Data curation, Writing - original draft.

**Kareem Salhiyyah:** Conceptualization, Resources, Software, Writing - review & editing, Project administration.

**Stephen Harding:** Resources, Software, Investigation, Formal analysis.

**Sunil Ohri:** Conceptualization, Funding acquisition, Writing - review & editing, Supervision, Validation.

## Registration of research studies

No registration required.

## Guarantor

Kareem Salhiyyah and Sunil Ohri.

## Provenance and peer review

Not commissioned, externally peer reviewed.
